# Kinetics of DNA looping by *Anabaena* sensory rhodopsin transducer (ASRT) by using DNA cyclization assay

**DOI:** 10.1038/s41598-021-03148-4

**Published:** 2021-12-09

**Authors:** Jae Jin Lee, Sung Hyun Kim, Keon Ah Lee, Kimleng Chuon, Kwang-Hwan Jung, Doseok Kim

**Affiliations:** 1 Department of Physics, Sogang University, Seoul, Korea; 2 Department of BioNanoScience Kavli Institute of Nanoscience of Delft University of Technology, Delft, The Netherlands; 3 Department of Life Science, Sogang University, Seoul, Korea

**Keywords:** Single-molecule biophysics, DNA-binding proteins

## Abstract

DNA cyclization assay together with single-molecule FRET was employed to monitor protein-mediated bending of a short dsDNA (~ 100 bp). This method provides a simple and easy way to monitor the structural change of DNA in real-time without necessitating prior knowledge of the molecular structures for the optimal dye-labeling. This assay was applied to study how *Anabaena* sensory rhodopsin transducer (ASRT) facilitates loop formation of DNA as a possible mechanism for gene regulation. The ASRT-induced DNA looping was maximized at 50 mM of Na^+^, while Mg^2+^ also played an essential role in the loop formation.

## Introduction

The interaction between protein and dsDNA is ubiquitous and plays an important role in living cells. The most popular example is a nucleosome in eukaryotic cell nucleus, where dsDNA is wrapped around histone proteins, and a modification of the histone or the dsDNA modulates the interaction between DNA and the histone as one of the mechanisms of epigenetics^1^. In prokaryotic cells, there are histone-like nucleoid-structuring (H-NS)^2,3^, heat-unstable (HU)^4^, and integration host factor (IHF)^5^ proteins that act like the histone. Proteins such as AraC^6^ and catabolite activator protein (CAP)^7^ bind to a specific sequence of dsDNA and bend it to suppress or promote a gene expression by changing the accessibility of the DNA region to RNA polymerase. Therefore, monitoring the structural change of the DNA is essential to understand the role played by the protein/DNA interaction.

Various experimental methods such as X-ray crystallography and NMR have been used to investigate the structural changes of dsDNA by proteins^8–12^**.** X-ray crystallography has been a golden standard tool to obtain detailed structural information of DNA with bound proteins^13^. Solution NMR method provides structural information obtained from a physiological buffer conditions^14^. Despite the detailed structural insights provided by these methods, the information is largely limited to the snapshot views of the protein-DNA complex and, hence, the actual reaction of the DNA can only be inferred.

Single-molecule fluorescence resonance energy transfer (FRET) has been successfully used to probe the structural changes of dsDNA induced by proteins as it measures accurately the change in the distance between two reporter dyes labeled on a dsDNA^15–19^. Advantage of smFRET over the other techniques, such as X-ray crystallography and NMR, is that it can measure the kinetics of binding and follow in-situ structural changes between the DNA and the protein. However, as FRET is limited by rather a short probing range (3 ~ 10 nm), experimental design (e.g. dye-labeling position) to bring the dye molecules in- and out of the probing range relies on a prior knowledge of the structure by NMR or X-ray crystallography. In this study, we demonstrate a smFRET-based assay that can monitor structural changes of dsDNA by proteins even without prior structural information.

**Figure 1:**
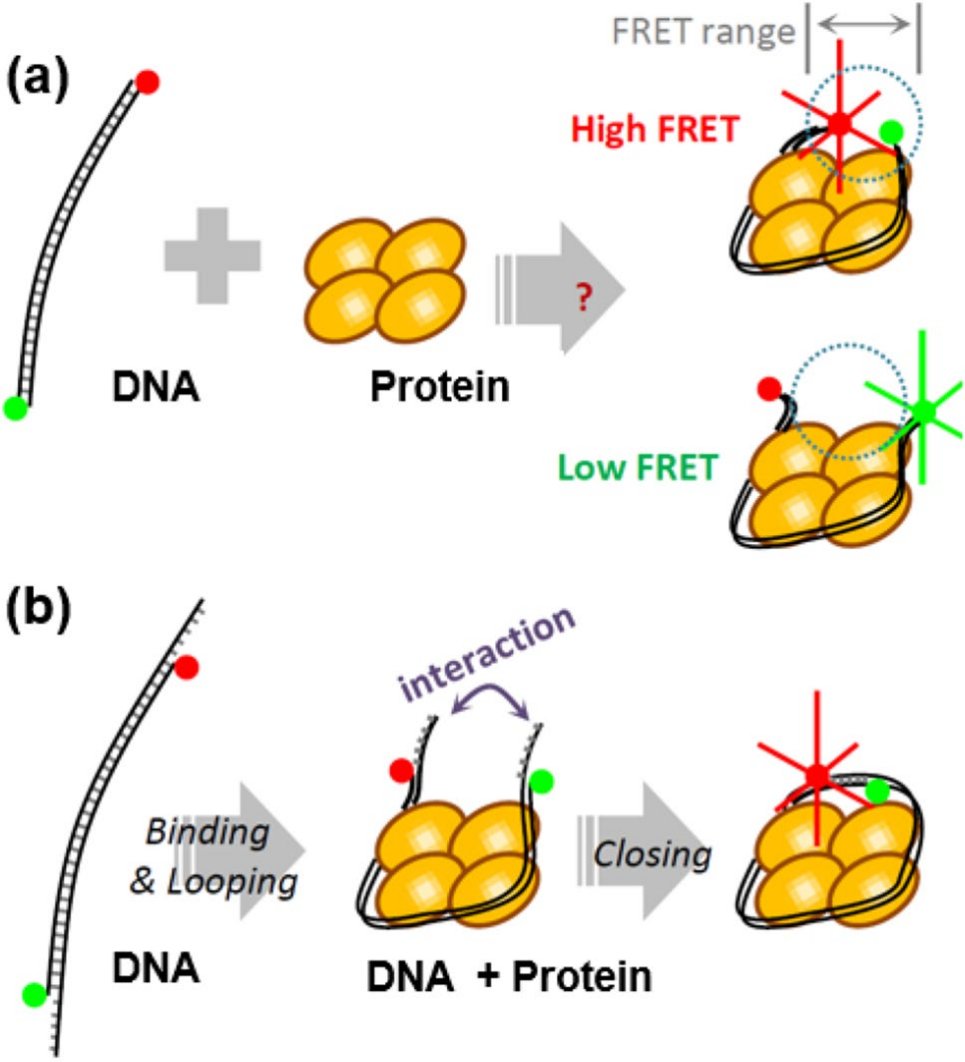
Schematic assay for the single-molecule cyclization (**a**) in the absence, and (**b**) presence of single strand (ss) tail. In (**a**), there is no guarantee to have high-FRET signal from the dye pairs labeled at the two ends of the dsDNA even if the dsDNA makes almost a close loop.

The single-molecule FRET-based cyclization assay is based on a single-molecule method originally developed to study the intrinsic bending properties of dsDNA^20,21^ and an ligation-based cyclization assay to observe the protein-induced DNA bending in ensemble level^22–24^. In case of protein-dsDNA studies via conventional smFRET, as illustrated in Fig. 1a, there is no guarantee of obtaining FRET signal as the two dye molecules may move away beyond the FRET probing range upon the structural change induced by the protein. To overcome this problem, a set of complimentary single-strand (ss)-overhangs can be attached at the ends of the dsDNA as shown in Fig. 1b. When dsDNA is bent in a ring form by a protein, the ss-overhangs at both ends would hybridize to each other and keep the two ends, thus the two dyes, in close proximity. This active locking mechanism by the ss-overhangs ensures a stable FRET signal and increases the effective probing range. Unlike the ligation-based cyclization assay, this FRET-based cyclization method does not require the helicity matching of the two dsDNA ends for ligation. Thus, the FRET-based cyclization method provides more accurate measure on the DNA looping kinetics.

In this study, we applied this FRET-based cyclization assay to study the bending of dsDNA by a protein called *Anabaena* sensory rhodopsin transducer (ASRT)^25^. ASRT is a 14-kDa soluble protein that is co-expressed with *Anabaena* sensory rhodopsin (ASR). ASR is a light-sensing dimeric and ASRT is a tetrameric protein in a cyanobacteria. ASRT interacts first with ASR at the cell membrane, and after being released from ASR, subsequently binds to dsDNA in the cytoplasm. It is suggested that the ASRT regulates several genes related to the light-harvesting system and the circadian clock^26^.

The detailed structure of ASRT was reported by X-ray crystallography and solution NMR studies^27,28^. It has been reported from EMSA and fluorescence correlation spectroscopy (FCS) that ASRT binds to the promotor region of the phycoerythrocyanin gene^29,30^. In particular, it showed high binding affinity in the region containing transcriptional and translation start sites. From these experimental results, a model was suggested that ASRT may regulate the gene expression via changing the structure of DNA, similar to CAP and AraC^25^. However, detailed structural changes of DNA by ASRT have not been reported.

We studied the structural changes of dsDNA by ASRT protein by using the single-molecule FRET-based cyclization assay. Upon binding of the ASRT, the two ends of the bent dsDNA came close, and the ss-overhangs hybridized with each other to form a stable ring shape. FRET changed readily upon introduction of ASRT in the buffer solution, indicating ASRT binding changed the structure of dsDNA to bend and wrap around the ASRT. By following the change in the FRET histogram, the looping kinetics was found to increase markedly by introducing ASRT into the buffer having Na^+^ and Mg^2+^ ions. This cyclization assay is a powerful tool for studying DNA–protein interaction and the subsequent structural change of the complex even when the binding structure is not well known a priori.

## Result

### DNA looping at 1 M NaCl buffer solution

To observe protein-induced DNA bending with the FRET-based cyclization assay, we designed a 100 bp-long DNA with 10 base-long ss-overhangs (Fig. 1b). Construction of the DNA and the detailed sequence is described in the methods. The sequences of the two ss-overhangs were complementary to each other such that the DNA molecule can be looped when the two ends are brought in close proximity (Fig. 1b). Upon looping, the donor (Cy3) and acceptor (Cy5) dyes, which were located at each end of the DNA, would report high FRET efficiencies. The length of the ss-overhangs was chosen to be 10 bases to have the looped circular form stable enough to be observed in our observation time (several tens of minutes), but not too stable to avoid self-assembly during the sample preparation. In physiological salt concentration, as the DNA was shorter than its persistence length (~ 50 nm)^31^, no looping, hence no FRET, would occur unless the linear DNA was significantly bent by a protein for example. Only at a high salt concentration, the dsDNA may spontaneously form a loop and give a high FRET value as reported previously^21,32^.

**Figure 2:**
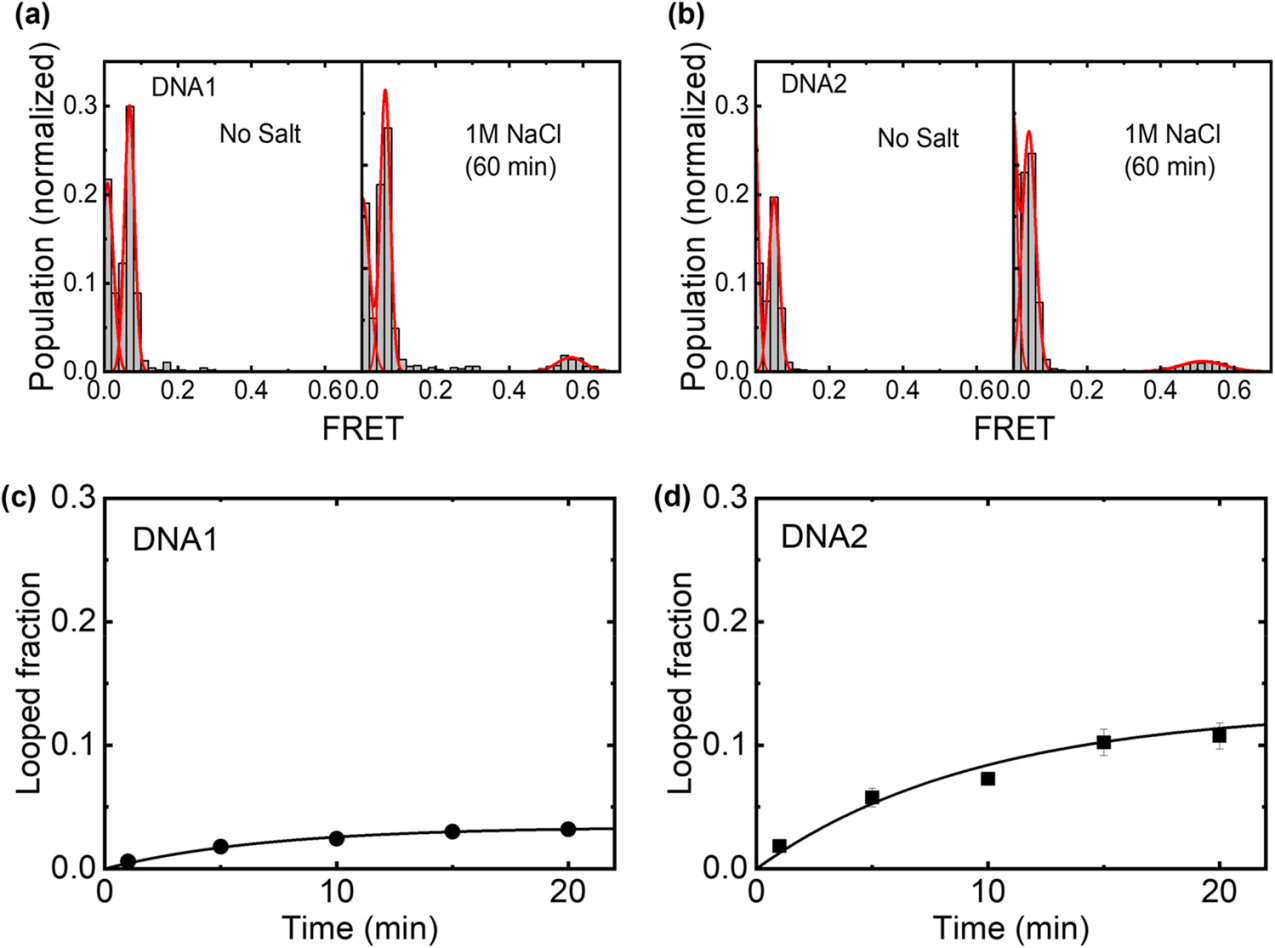
DNA looping induced by high concentration of salt (1 M NaCl). Single-molecule FRET histograms of (**a**) DNA1 and (**b**) DNA2 obtained after incubation (left) with and (right) without 1 M NaCl. (**c**, **d**) Time courses of the fraction of looped DNA for (**d**) DNA1 and (**d**) DNA2.

To check if our DNA would indeed show high FRET value upon looping, we first examined it with high concentration of NaCl. The DNA was immobilized on a bovine serum albumin (BSA)-coated quartz glass surface via biotin-streptavidin linker. Under 532 nm laser excitation, fluorescence intensities of individual Cy3 and Cy5 dyes were recorded separately to yield FRET efficiencies of the individual DNA molecules. Initially, with a buffer solution carrying no salt, we observed two FRET populations at *E* ~ 0 and *E* ~ 0.05 (Fig. 2a, left panel). By directly exciting the acceptor dyes, we identified that the population at *E* ~ 0 was DNA molecule labeled only with Cy3, and the *E* ~ 0.05 was both Cy3 and Cy5 are labeled on the DNA (Figure S1). Although the expected FRET value was 0 with the dye separation of 100 bp (~ 34 nm in a B-form DNA configuration), the observed FRET value was E ~ 0.05 due to the direct excitation of Cy5 by the 532 nm laser (Figure S1). The same two peaks at *E* ~ 0 and *E* ~ 0.05 (Fig. 2b, left panel) were also observed from the same DNA construct but carrying a different sequence (hereafter referred as DNA1 and DNA2 for the two sequences, see methods for detail).

When we introduced 1 M NaCl to the sample chamber, a population at *E* ~ 0.55 appeared in the histogram due to cyclization of the DNA similar to the previous studies (Fig. 2a-b, right panel)^21,32^. The fraction of the looped DNA was quantified by fitting the histogram with Gaussian functions. The looping kinetics were characterized by a single exponential function (**Eq. ****3** in methods) (Fig. 2c-d). DNA1 showed a looped fraction value of 0.04, and the looping and unlooping rates were 0.0048 min^−1^, and 0.18 min^−1^, respectively. DNA2 showed looped fraction 0.12, looping rate 0.013 min^−1^, and unlooping rate 0.09 min^−1^. The two DNA samples showed different looping fractions and rates, presumably due to the difference in the intrinsic curvature and flexibility of the two DNA^21^. With a similar cyclization assay in 1 M NaCl condition, Vafabakhsh and Ha reported a looped fraction of ~ 0.8 in and the looping rate of ~ 0.15 min^−1^. Both values are appreciably larger than our values, presumably due to the position of DNA immobilization as well as the presence of a 1 base gap by the two sticky ends (see Discussion for details)^33^.

### Looping fraction of DNA induced by ASRT

As our DNA constructs were shown to report high FRET upon cyclization, we then attempted to monitor bending of the DNA induced by a protein. As a test protein, we chose ASRT of which DNA binding activity was reported but lacking detailed information on the structure of the bound DNA^28,30^. As a signal transducer protein which binds to DNA for gene regulation^29^, we hypothesized that ASRT might induce significant structural change of DNA, similar to many other gene regulation proteins^2–7^. Then our FRET-based cyclization assay may report the structural change in the DNA.

To observe ASRT-induced DNA bending, excess amount of ASRT (100 μM) was put into the sample chamber and the changes in the FRET value of individual DNA was monitored. We used the salt condition of 50 mM NaCl which was used in the previous FCS and NMR studies for ASRT-DNA binding^28,30^. Note that 50 mM NaCl was too weak to induce spontaneous DNA bending (Figure S2, upper left panel). The concentration of ASRT (100 μM) in substantial excess over its *K*_D_ (1.7 ± 0.2 μM) was to ensure that any slow structural change of DNA can also be observed within our observation time^28^. However, no meaningful change was seen in the FRET histogram even after 60 min incubation of DNA with ASRT (Figure S2), suggesting that ASRT binding to the DNA is not able to induce DNA bending in the buffer condition tested above.

**Figure 3:**
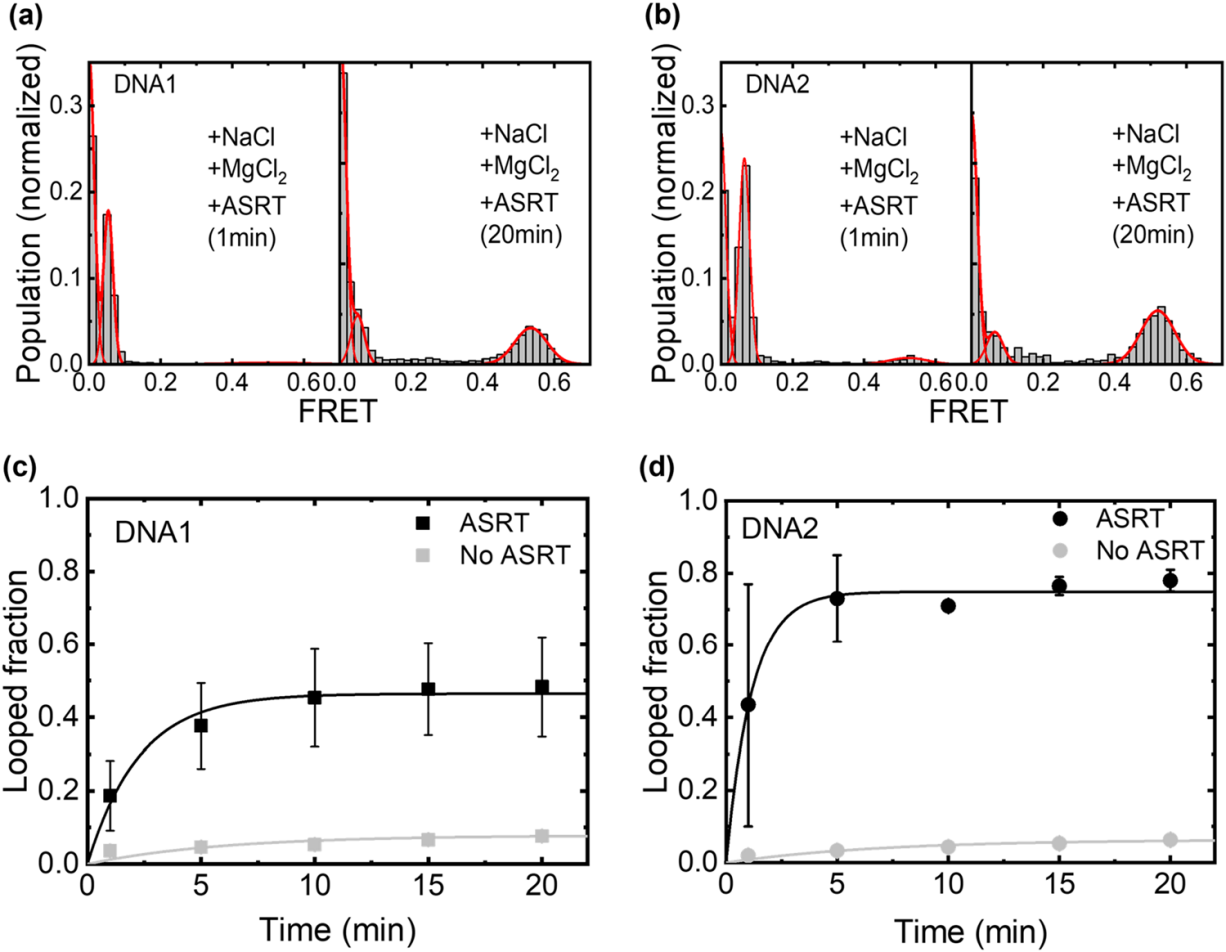
Single-molecule FRET histograms in the presence of ASRT with (**a**) DNA1 and (**b**) DNA2. Data obtained (left) right after- and (right) 20 min after injection of buffer containing ASRT, 10 mM MgCl_2_ and 50 mM NaCl. (**c**, **d**) The fraction of the looped DNA molecules obtained from the FRET distributions. (**c**) for DNA1 and (**d**) for DNA2.

Interestingly, to our surprise, we found that addition of 10 mM MgCl_2_ in the reaction buffer together with NaCl (50 mM) allowed ASRT to induce cyclization of the dsDNA, showing clear peak at *E* ~ 0.55 in the FRET histograms (Fig. 3a,b). For quantitative comparison of the ASRT-induced DNA bending, we measured the time courses of the FRET changes after injection of ASRT into the chamber and analyzed them with a simple kinetic model (Fig. 3c,d**,** the fit results with Eq. 3 are summarized in Table 1, see methods for details). Faster DNA looping rates with ASRT (0.20 ± 0.036 min^−1^ for DNA1 and 0.65 ± 0.078 min^−1^ for DNA2) were seen as compared to that observed with 1 M NaCl in Fig. 2a-d. The looped fractions (0.48 and 0.78 for DNA1 and DNA2, respectively) were also substantially larger. By contrast, in the absence of ASRT, no changes in FRET histograms were observed at the same concentrations of NaCl and MgCl_2_, confirming that the DNA cyclization is indeed induced by ASRT. Our result clearly shows ASRT is capable of bending the DNA, and Mg^2+^ ion is required for this structural manipulation of DNA. As a control experiment, 100 μM of BSA (bovine serum albumin) was put into the same buffer instead of ASRT. No change in FRET was seen even after 60 min (Figure S3), demonstrating the structural change of the DNA was specific to ASRT.

**Table 1:** looping fraction and rate of DNA induced by ASRT.

	Looped Fraction (at 20 min) with ASRT	Looped Fraction (at 20 min) no ASRT	Looping rate (min^−1^) with ASRT	Looping rate (min^−1^) no ASRT	Unlooping rate (min^−1^) with ASRT	Unlooping rate (min^−1^) no ASRT
DNA1	0.48 ± 0.095	0.087 ± 0.0058	0.20 ± 0.036	0.013 ± 0.0070	0.24 ± 0.041	0.16 ± 0.079
DNA2	0.78 ± 0.030	0.067 ± 0.015	0.65 ± 0.078	0.0091 ± 0.035	0.22 ± 0.029	0.13 ± 0.049

### Salt concentration dependence

While previous studies reported interaction between ASRT and DNA in a buffer without divalent ions^28,30^, our cyclization assay showed that Mg^2+^ was essential in the ASRT-induced DNA bending. In other words, ASRT may bind to DNA in the absence of Mg^2+^, yet Na^+^ alone cannot trigger the ASRT-induce DNA bending. Here, we questioned if the role of Na^+^ is limited only to the binding process or if it also plays an additional role in bending in cooperation with Mg^2+^.

**Figure 4:**
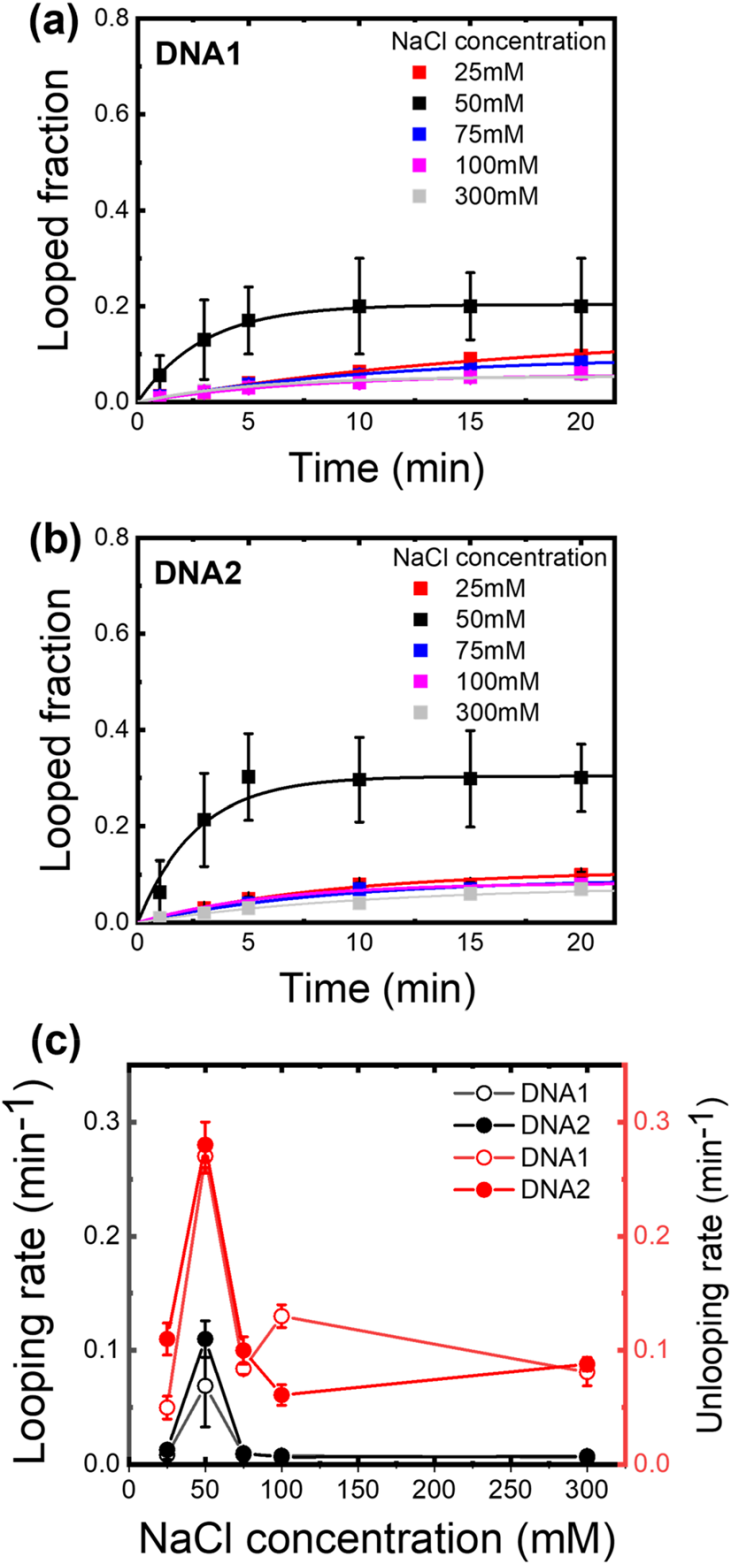
Salt concentration dependent of DNA looping fraction induced by ASRT. (**a**) DNA1. (**b**) DNA2. (**c**) looping (black color) and unlooping (red color) rates of DNA1 (open circles) and DNA2 (filled circles) according to NaCl concentration. In the experiment, the concentration of NaCl was changed to 25 ~ 300 mM. The concentration of MgCl_2_ was fixed to 10 mM. The right panel shows the unlooping rate. Error bars are standard errors from three independent experiments.

To check for this idea, we repeated the measurements at various concentrations of NaCl with MgCl_2_ concentration fixed at 10 mM. The Mg^2+^ ion concentration is in a physiologically relevant range as the total magnesium concentration in various cells ranges from 5 to 30 mM^34^, and a nuclease from *Anabaena* sp. strain PCC 7120, for example, requires Mg^2+^ as a cofactor and functions optimally at 5 mM Mg^2+^ concentration^35^. Interestingly, the maximum faction of looped DNA after 20 min incubation with ASRT was highest at 50 mM NaCl concentration and decreased at higher concentrations in both cases of DNA1 and DNA2 (Fig. 4a,b, Tables 2, 3). The enhanced looping at 50 mM NaCl was mainly due to the relatively large change in the looping rates than the unlooping rates (Fig. 4c). The decrease in the looping rate at higher NaCl concentrations confirmed that the cyclization process was indeed induced by the protein, not by the salt as observed in Fig. 2a,b. In particular, at 300 mM NaCl, the looped fraction and looping rate were almost the same as those in the absence of ASRT, which shows that ASRT is inactive at such a high NaCl concentration. When DNA1 and DNA2 were compared, we noted that only the looping rates, not the unlooping rates, were affected by the DNA sequence (Fig. 4c and Table 1).

**Table 2:** Salt concentration dependent of DNA1-ASRT interaction.

DNA1	Looped Fraction (at 20 min) with ASRT	Looping rate (min^−1^) with ASRT	Unlooping rate (min^−1^) with ASRT
25 mM NaCl	0.097 ± 0.0058	0.0085 ± 0.0041	0.05 ± 0.01
50 mM NaCl	0.20 ± 0.10	0.069 ± 0.036	0.27 ± 0.015
75 mM NaCl	0.083 ± 0.0061	0.0089 ± 0.0050	0.084 ± 0.005
100 mM NaCl	0.060 ± 0.0090	0.0078 ± 0.0023	0.13 ± 0.01
300 mM NaCl	0.057 ± 0.0050	0.0063 ± 0.00040	0.081 ± 0.012

**Table 3:** Salt concentration dependent of DNA2-ASRT interaction.

DNA2	Looped Fraction (at 20 min) with ASRT	Looping rate (min^−1^) with ASRT	Unlooping rate (min^−1^) with ASRT
25 mM NaCl	0.10 ± 0.0060	0.013 ± 0.0030	0.11 ± 0.014
50 mM NaCl	0.30 ± 0.070	0.11 ± 0.016	0.28 ± 0.020
75 mM NaCl	0.081 ± 0.0059	0.01 ± 0.0030	0.10 ± 0.012
100 mM NaCl	0.080 ± 0.0051	0.0066 ± 0.0010	0.061 ± 0.009
300 mM NaCl	0.070 ± 0.0045	0.0073 ± 0.0070	0.088 ± 0.006

**Figure 5:**
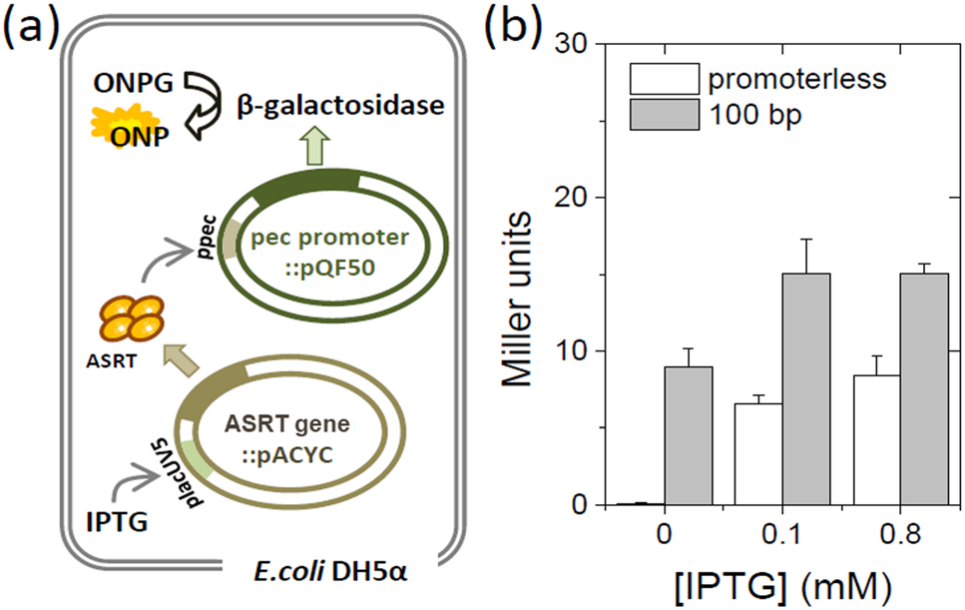
(**a**) Schematic of the vector constructs for β-galactosidase assay (**b**) Comparison of the expression level of β-galactosidase in Miller units in the absence of promoter (QF50, white bars) and 100 bp *pec* promoter (gray bars) inserted into the vector at two different induction conditions. Error bar are standard errors from three independent experiments.

As loop formation of DNA is often found in other regulatory processes, we wished to examine that the DNA binding and looping ability of ASRT indeed plays a role in the gene regulation in vivo. To determine the downstream gene regulation by ASRT, we carried out the β-galactosidase assay. The lacZ gene of which the promoter region was replaced by the same sequence of 100 bp dsDNA used in the cyclization assay was transformed into *E. coli* cell (Fig. 5a). ASRT regulated by lacUV5 promoter, which is inducible with isopropyl β-D-1-thiogalactopyranoside (IPTG), was also transformed into the *pec* promoter contained cell. Promoter-less lacZ construct was used as a negative control. The activity was determined through the spectral change of substrate ortho-Nitrophenyl-β-D-galactoside (ONPG) (Fig. 5b). After induction of ASRT protein at two different IPTG concentrations (0.1 and 0.8 mM), the activity of β-galactosidase was increased in all constructs with different efficiency, which implies this reporter gene expression was caused by the presence of ASRT. This result is in consistent with the previous study in which the similar regulatory role of ASRT on the *pec* promoter-controlled reporter gene expression was observed upon IPTG induction in the presence and absence of ASR operon^29^.

## Discussion

DNA in the double stranded B-form structure shows a strong rigidity due to its long persistence length (~ 50 nm in physiological salt condition). The structural manipulation of dsDNA is, therefore, one of the important tasks in the cell for the gene regulation, replication, and genome packaging. In this study, we showed that the single-molecule FRET cyclization assay is an easy and powerful method for monitoring the protein-induced DNA bending. We successfully demonstrated this new approach for studying ASRT-DNA interactions and explored its possible regulatory role in the gene expression.

In our FRET-based cyclization assay with 1 M NaCl, 4% and 13% of DNA1 and DNA2 were looped, respectively (Fig. 2c,d). These looping fractions are considerably smaller than the values reported by Vafabakhsh and Ha tested under the same salt conditions^21^. The major differences are the immobilization position of the DNA to the surface and the presence of 1 base gap between the sticky ends and dsDNA body. In the previous report, the center of DNA was immobilized at the substrate surface, while the end of the dsDNA was immobilized at the surface in the current experiment. According to the theoretical and numerical results by Waters and Kim^36^, the DNA immobilized at the center has a looping fraction value more than 5 times higher than the DNA immobilized at the end. In addition, Jeong and Kim experimentally showed that the base stacking interaction at a nicked junction of DNA contributes to the stability of the looped DNA significantly^33^. Hence, the presence of the 1 base gap in our DNA design would further destabilize the looped form. These two factors can qualitatively account for the difference between the current result and the previous report. While the relatively small looping fraction did not obstruct the detection of the protein induced bending in this study, an optimized design for the gap and the immobilization scheme tailored for each target protein may potentially improve the result. For example, giving a longer linker between the dsDNA and the biotin for surface attachment would give more room for a bulky protein to interact with the DNA. A longer gap, i.e. longer linker between the dsDNA and the complementary sequences in the ss-overhangs, would give a better chance for the ss-overhangs to find each other, allowing a detection of a less bent conformation.

Previously, the binding between ASRT and DNA was investigated using various experimental methods such as NMR^28^, Electrophoretic Mobility Shift Assay (EMSA)^29^, and Fluorescence correlation spectroscopy (FCS)^30^. The NMR study found that three amino acid residues (R109, N113, Q110) in the C-terminal of ASRT are the main contact points with the DNA^28^. By using EMSA, it has been shown that multiple regions in the promoter region and transcription start sites of the phycoerythrocyanin gene have higher binding affinity with ASRT^29^. The FCS study^30^ found a *K*_D_ of ~ 10 μM between ASRT and 20 bp fragment of phycoerythrocyanin promotor region and reported a weak sequence dependent changes of the *K*_D_. Together, a hypothesis emerged: ASRT might induce changes in the structure of DNA, such as in the cases of CAP and AraC proteins, for gene regulation purpose. However, the above-mentioned techniques were unable to check if ASRT induces large structural change of the bound DNA. Our FRET-based cyclization assay observed that 40% of DNA1 and 80% of DNA2 form a loop around ASRT. The loop was formed within 5 min, showing that ASRT readily changed the structure of a rigid DNA into a form wrapping around the ASRT. (Fig. 2). Interestingly, DNA2, having a computer-generated random sequence, showed higher looping fraction and faster looping rate compared to DNA1, a promoter fragment of phycoerythrocyanin gene. While it is unclear which hidden feature in the random sequence of DNA2 confers such enhancement, this sequence dependence of ASRT reflects its role in gene regulation, which is remained for the future study.

The binding and looping of DNA by ASRT was found to be maximized at around 50 mM Na^+^ concentration (Fig. 4). By contrast, the looping under the high Na^+^ concentration of 300 mM was very small, almost the same as in the case of no-ASRT condition (Tables 1, 2, 3). Interestingly, the Na^+^ concentration in *Anabaena* is about 50 mM, and regarding the fact that the *Anabaena* was found and grows better in fresh water, it is tempting to speculate that this salt-dependent functionality of ASRT is a result of coping in its native environment^37^. In addition to Na^+^ ion, we found that Mg^2+^ ion is required for bending of DNA in the DNA/ASRT complex (Figure S2). As the previous studies on the DNA/ASRT interaction were carried out in NaCl-only conditions^28–30^, it is natural to think that Na^+^ is involved in the binding of the ASRT to DNA and Mg^2+^ ions are essential in the structural stability of DNA.

Our galactosidase assay shows that ASRT has an ability to regulate gene expression (Fig. 5). The basal level of β-galactosidase activity was nonzero in the cell even in absence of ASRT, possibly due to the influence of the transcription machinery of the host cell. However, despite the high basal level, we observed notable increment of ASRT mediated activity, confirming that the ASRT changes the gene expression in heterologous expression system. This result, together with our single-molecule FRET based cyclization assay, supports the idea of the regulatory role of ASRT for the genes related to the light-harvesting systems in response to the environmental light quality detected by the rhodopsin on the membrane.

In conclusion, we demonstrated that the single-molecule cyclization methods can be used to monitor the protein-aided loop formation of short dsDNA fragments. The method provides a simple strategy to study the structural change of DNA upon binding of protein of which the detailed structural information is not available. We hope that the method described is applicable to other protein-aided bending and looping processes such as in nucleosome dynamics or in transcription pre-initiation complexes.

## Methods

### DNA design for single molecule cyclization assay

To probe the looping of the dsDNA around ASRT, we prepared two dye-labelled dsDNA constructs with 10 base-long single strand overhangs by annealing and ligating four synthesized ssDNA strands (IDTDNA) to get the 101 bp-long DNA. Overhang length of 10 nt was chosen to maintain a stable loop once a loop was formed by ASRT-DNA interaction^21^. One sequence (DNA1) was adopted from the promoter region of the phycoerythrocyanin (*pec*) gene. This gene is involved in the regulation of photosynthesis in cyanobacteria, and can be a target of ASRT in the signal transduction pathway^29^. A donor (Cy3) and an acceptor (Cy5) dye were labelled at the ends of this dsDNA (Figure S1). The sequence of the other (DNA2) is generated by a custom-built Matlab code) (Figure S1).

The DNA1 and DNA2 are made as follows. A total of four short ssDNAs were purchased to make a 101 bp DNA sample to be used in the experiment. (Integrated DNA Technologies, Inc) The ssDNA sequence used to make DNA1 is as follows. 5′-/Biotin/ CAG AAT CCG TGA ATA TTT GTT TTC TAA ATA GTA AGA ATA ATT GCA ATC GAC CTT ATA AAA AGC TGC AAT GAC CTT TAG GAG -3′ (81 bases), 5′-/Phosphorylation/ GAA AGA AAG ATG CTC GAT GCT TTT TCC AAA /cy3/ -3′ (30 bases), 5′-ACG GAT TCT GTT TTG GAA AAA GCA TCG AGC ATC TTT CTT TCC TCC TAA AGG TCA TTG CAG CTT TTT ATA AGG TCG ATT GCA A -3′ (82 bases), 5′-/Phosphorylation/ TTA TTC TTA CTA TTT AGA AAA CAA ATA TTC /cy5/ -3′ (30 bases). After mixing the four strands, the temperature was raised to 90 degrees and then slowly cooled for annealing. The dsDNA produced afterwards has kinks in two places, which were ligated at 16 degrees for 8 h using the T4 DNA ligase kit (New England Biolabs). The ligated product was purified by 15% SDS-PAGE gel, Finally, a dsDNA sample having a length of 101 bp was obtained by re-annealing the gel elution. DNA2 was made in the same process, and the sequence of ssDNA is as follows. 5`-/Biotin/ CAG AAT CCG TTC TGT GAC TGG TGA GTA CTC AAC CAA GTC ATT CTG AGA ATA GTG TAT GCG GCG ACC GAG TTG CTC TTG CCC -3`(81 bases), 5′-/Phosphorylation/ GGC GTC AAC ACG GGA TAA TAC CGC GCC ACA /Cy3/ -3`(30 bases), 5′-ACG GAT TCT GTT GTG GCG CGG TAT TAT CCC GTG TTG ACG CCG GGC AAG AGC AAC TCG GTC GCC GCA TAC ACT ATT CTC AGA A -3′ (82 bases), 5′-/Phosphorylation/ TGA CTT GGT TGA GTA CTC ACC AGT CAC AGA /cy5/ -3′ (30 bases).

### *Anabaena* sensory rhodopsin transducer preparation

Overnight cultures of *E. coli* BL21 that contain the plasmid for the production of his-tagged ASRT proteins were induced with 0.8 mM IPTG (Duchefa, Netherland) for 4 h at 35 ℃. Induced *E. coli* cells were harvested and suspended in sonication buffer (50 mM TrisHCl pH 7.0, 150 mM NaCl). Cells were lysed with 0.5 mM PMSF (phenylmethylsulphonyl fluoride, USB, USA) by sonication (Branson sonifier 250) at 4 ℃ followed by low-speed (3,220 × g for 20 min) centrifugation (Eppendorf centrifuge 5810R) to remove cell debris. Cell lysates were then sedimented at 95,000 × g for 1 h at 4 ℃ (Ti70 rotor, Beckman XL-90 ultracentrifuge), and the supernatant was transferred to a new tube. The supernatant was incubated with Ni^2+^-NTA agarose (Qiagen) and purified with imidazole^38^.

### Single molecule imaging of the DNA looping

For the single-molecule DNA cyclization assay, we used a homebuilt total internal reflection microscope. The dye-labelled DNA was immobilized on a bovine serum albumin coated quartz glass surface via biotin-streptavidin linker. To increase photo-stability of the dyes for fluorescence measurements, we added 1 mg/ml glucose oxidase (Sigma), 0.8% (w/v) dextrose (Sigma), 0.04 mg/ml catalase (Sigma), and 2 mM 6-hydroxy-2,5,7,8-tetramethylchroman-2-carboxylic acid in the solution. The dyes were illuminated by the 532 nm Laser beam (CrystaLaser) via total internal reflection. The fluorescence data were collected by a microscope objective lens (Olympus, NA1.2) and spectrally divided into Cy3 and Cy5 channel by a dichroic mirror (Omega Optical) before imaging on an EMCCD camera (Andor technology). To extract the fluorescence intensity time trace of individual molecules, the collected fluorescence image was analyzed by home-built software written in IDL and Matlab.

Practically, a crosstalk between donor and acceptor channel should be considered due to the spectral overlap between the donor and acceptor fluorescence. Hence, the FRET efficiency can be now calculated by

1 $$ {\text{E}} = \frac{{{\text{F}}_{{\text{A}}} - L \times F_{D} }}{{F_{A} + F_{D} }}, $$

where L is the leakage correction factor to compensate for the donor fluorescence photons entering the acceptor channels. To build a single-molecule FRET histogram, we averaged the first 10 data points (each data points corresponds to 0.1 s exposure) from the time traces of the individual molecules and calculated the individual FRET efficiencies by using Eq. (1).

This FRET signal should reflect the structural change of single DNA construct upon binding with ASRT. DNA molecules are immobilized on the glass surface with distance at least several micrometers apart from each other. Afterwards, a fresh buffer solution flows to the channel to flush out the free DNAs (not immobilized on the surface) before adding ASRT to the channel. Therefore, the observed FRET signal can only be caused by an ASRT with single DNA complex.

### Calculating looping and unlooping rates from histograms

We observed that fraction of looped molecules over time obtained from histograms followed the exponential decay curve of the form ${\text{A}}\left( {1 - {\text{e}}^{{ - {\text{B}} \cdot {\text{t}}}} } \right)$ where A and B are fitting value. This equation can be obtained by assuming a system consisting of unlooping and looping state^21^.

2 $$ {\text{ Unlooped }}\left( {\text{U}} \right){ }\begin{array}{*{20}c} {k_{l} } \\ \rightleftarrows \\ {k_{u} } \\ \end{array} {\text{ Looped }}\left( {\text{L}} \right), $$

where ${\text{k}}_{{\text{l}}}$ and ${\text{k}}_{{\text{u}}}$ are looping and unlooping rates.

The fraction of looped molecules is expressed as follows.

3 $$ \frac{\left[ L \right]}{{\left[ L \right] + \left[ U \right]}}\left( t \right) = \frac{{k_{l} }}{{k_{l} + k_{u} }}\left( {1 - {\text{e}}^{{ - \left( {{\text{k}}_{{\text{l}}} + k_{u} } \right)t}} } \right), $$

where [L] is the area obtained by Gaussian curve fitting to the high FRET population (E ~ 0.55), [U] is the area obtained by Gaussian curve fitting at a low FRET (E ~ 0.05), and [L] / ([L] + [U]) is fraction of looped molecules.

From this model, we can determine the unlooping rate and looping rate through fitting.

4 $$ {\text{k}}_{{\text{l}}} = A \times B,{\text{ k}}_{{\text{u}}} = B \times \left( {1 - A} \right), $$

where $${\text{A}} = k_{l} /\left( {k_{l} + k_{u} } \right)$$ and $${\text{B}} = {\text{k}}_{{\text{l}}} + k_{u}$$.

### Beta-galactosidase assay

Overnight-cultured *E. coli* DH5α cells containing two plasmids with appropriate promoter related lacZ gene and ASR whole operon were induced at different concentrations of Isopropyl β-D-1-thiogalactopyranoside (IPTG) (0.1 mM – 0.8 mM) for 4 h at 35 ℃. After measuring OD at 600 nm, 1 mL of cultured cells were harvested and suspended in 600 μl sonication buffer (50 mM TrisHCl pH 7.0 and 150 mM NaCl). Sonication was performed for 10 min (30 s pulse and 30 s rest). The reaction was started by mixing 500 μL cell lysate into the 4.5 ml preincubated buffer (0.1 M sodium phosphate pH 7.0 and 0.16 mg/ml ortho-Nitrophenyel-β-D-galactoside (ONPG)) at 30 ℃ and was stopped after 10 min of incubation by mixing 800 μl reaction mixture with 200 μl of 1 M Na_2_CO_3_. Afterwards, the visible absorption was measured at 420 nm (OD_420_) using 0 min sample as base. Miller unit was calculated from OD_600_ and OD_420_ values.

## Supplementary Information

Supplementary Information.
