# Supplementary material for: Kinetics of DNA looping by Anabaena sensory rhodopsin transducer (ASRT) by using DNA cyclization assay
Source: Sci Rep. 2021 Dec 9;11:23721. doi: 10.1038/s41598-021-03148-4 (PMC8660804; doi:10.1038/s41598-021-03148-4)
Supplement: Supplementary file 1 — Supplementary Information. [file 41598_2021_3148_MOESM1_ESM.docx]

**Kinetics of DNA looping by *Anabaena* sensory rhodopsin transducer (ASRT) by using DNA cyclization assay**

Jae Jin Lee^1^, Sung Hyun Kim^2^, KeonAh Lee^3^, Kimleng Chuon^3^, Kwang-Hwan Jung^3^, and Doseok Kim^1*^

**^1^**Department of Physics, Sogang University, Korea,

**^2^**Department of BioNanoScience Kavli institute of Nanoscience Delft University of Technology, Netherlands,

**^3^**Department of Life Science, Sogang University, Korea.

* Corresponding Author

Doseok Kim

Department of Physics, Sogang University, Korea

Tel: +82 2 705 8878

Fax: +82 2 711 4518

E-mail: doseok@sogang.ac.kr

**Supporting Information**

**S1. Examination of the labeled DNA by direct excitation of the dyes**

Alternating-laser excitation method was used to check the fluorescence-labeled double dsDNA prepared by hybridization and ligation of four single stranded DNAs. The dsDNA was illuminated by 532 nm laser beam to excite Cy3 dyes and the fluorescence signals from Cy3 and Cy5 emission channels ($F_{D}^{G}$ and $F_{A}^{G}$, respectively) were separately measured to obtain FRET efficiency. Then, 532 nm laser was turned off and 633 nm laser was turned on to measure the fluorescence of Cy5 dyes, $F_{A}^{R}$, by direct excitation. A 2-dimensional histogram with FRET efficiency and stoichiometry parameter, $S=(F_{D}^{G}+F_{A}^{G})/(F_{D}^{G}+F_{A}^{G}+F_{A}^{R})$, was built to identify the DNA molecules labeled with both Cy3 and Cy5. In the 2D histogram (**Fig. S1 (a) and (b)**), two peaks were appeared one of which was donor only molecules (*E* ~ 0, *S* ~ 0.1) and the other of which was the molecules labeled with both donor and acceptor (*E* ~ 0.05, *S* ~ 0.5). Because Cy3 and Cy5 were located at the opposite ends of the DNA (**Fig. S1 (a) and (b)**), only the properly hybridized DNA molecules can have both dyes.

**
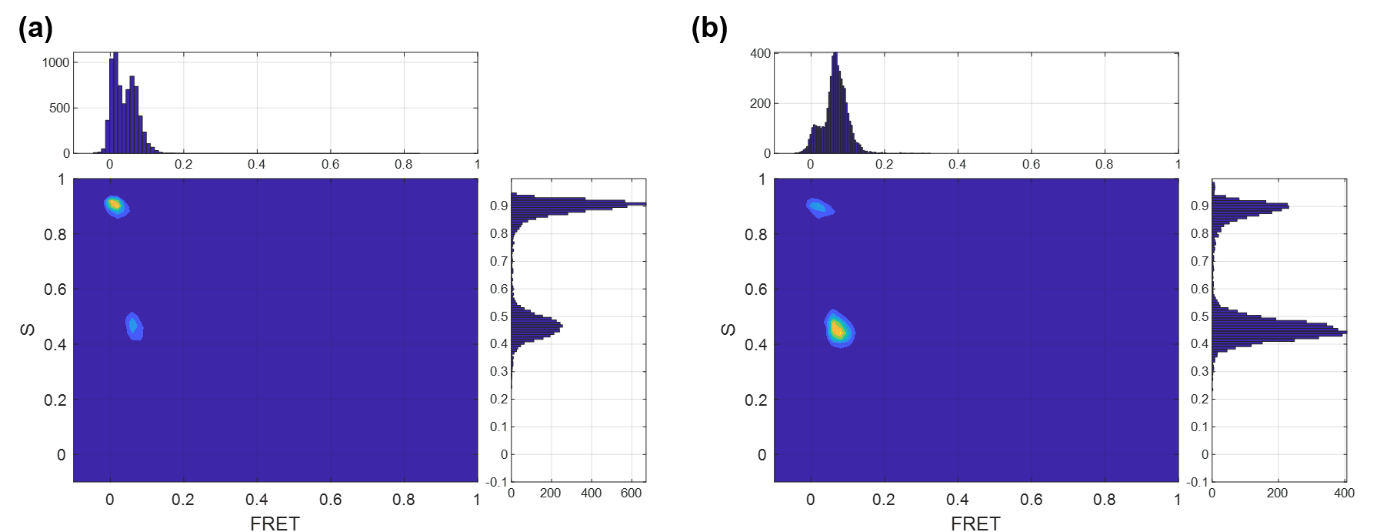
**

**Figure S1. (a)** DNA1 : Histograms of single-molecule FRET (*E*, top panel) and stoichiometry parameter (*S*, right panel) with their correlation plots (middle) obtained in the absence of salt and ASRT. The stoichiometry parameter, *S* (right), allowed us to distinguish the two peaks appeared in the single-molecule FRET distribution (top): one was donor only molecules (*E* ~ 0, *S* ~ 0.1) and the other was the molecules labeled with both donor and acceptor (*E* ~ 0.05, *S* ~ 0.5). (b) DNA2

**S2. ASRT-DNA binding in the absence of Mg^2+^ ion**


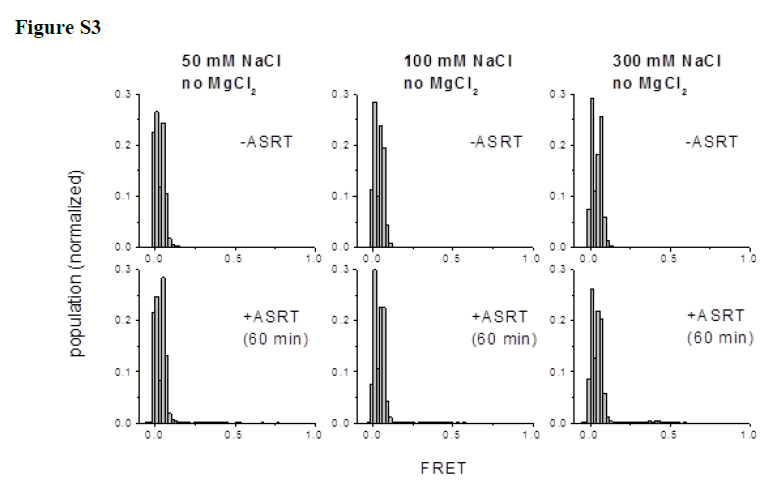


**Figure S2.** Single-molecule FRET histogram obtained at various NaCl concentrations without MgCl2 in the absence- (top panels) and presence of ASRT (bottom panels, 60 min incubation). Even with 60 min incubation with ASRT, we did not observe any meaningful changes in the looped DNA population at E~0.5.

**Figure S2.** Single-molecule FRET histogram obtained at various NaCl concentrations without MgCl_2_ in the absence (top panels) and presence of ASRT (bottom panels, 60 min incubation). Even with 60 min incubation with ASRT, we did not observe any meaningful changes in the looped DNA population at *E* ~ 0.5.

**S3. BSA-DNA binding experiment**


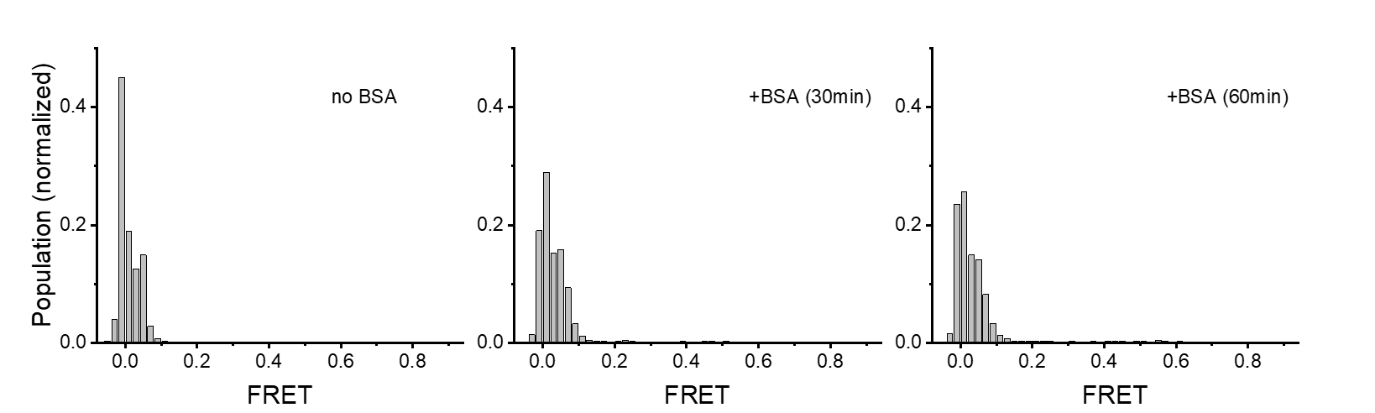


**Figure S3.** Single-molecule FRET histogram of DNA1 with Bovin serum albumin (BSA). DNA bending did not occur even after 60 minutes after 100uM BSA was added. The salt concentration conditions are 50mM NaCl, 10mM MgCl_2_.
